# Theoretical investigation of the interaction between the metal phthalocyanine [MPc]a(M = Sc, Ti, and V; a = –1, 0, and +1) complexes and formaldehyde

**DOI:** 10.3906/kim-2006-12

**Published:** 2021-02-17

**Authors:** Nasim HASSANI

**Affiliations:** 1 Department of Chemistry, College of Sciences, Shiraz University, Shiraz Iran

**Keywords:** Metal phthalocyanine, formaldehyde, electronic properties, HOMO-LUMO energy gap, gas detection, DFT

## Abstract

Formaldehyde (FA, CH_2_O) is one of the toxic volatile organic compounds that cause harmful effects on the human body. In this work, the interaction of FA gas with metal phthalocyanine (MPc) molecules was studied by employing density functional theory calculations. A variety of [MPc]^a^ (M = Sc, Ti, and V; a = –1, 0, and +1) complexes were studied, and the electronic properties, interaction energies, and charge transfer properties of all of the studied molecules were systematically discussed. Among the studied complexes, the Sc and Ti phthalocyanines were more reactive toward the adsorption of FA gas. Moreover, it was revealed that the interaction of the [ScPc]^+1^ and [TiPc]^0^ complexes with the CH_2_O molecule was stronger, in which the highest occupied molecular orbital (HOMO) and lowest unoccupied molecular orbital (LUMO) energy gap of 46% and 36% decreased after FA adsorption. The results indicated that the MPc-based materials may be a promising candidate for the detection of FA gas.

## 1. Introduction

Formaldehyde (FA, CH_2_O) is a toxic gas that is exposed to indoor air through manufacturing processes [1,2]. Increasing the concentration of FA in the air leads to an adverse health effects on humans and animals. Consequently, to accurately control of the concentration of FA in ambient air, it is urgent that new sensing material for detection of the FA is developed.

During recent last decades, various materials have demonstrated the potential to become a chemical sensor, such as carbon nanotube [3], the nanowire of various materials [4–8], nanoparticle arrays [9], mono-layer capped metal nanoparticles [10], metal oxides [11]. Sensors based on metal phthalocyanines (MPcs) are one of the best materials available to detect gases, because they offer a small-size, high-sensitivity, low-cost, ease of synthesis, and low processing temperature [12–14]. In addition, MPc aromatic macrocycles have the ability to stack and form crystalline and poly-crystalline films, which make these molecules appropriate for the design of field-effect transistors [15,16]. Furthermore, MPc-based sensors have shown strong absorption in ultraviolet-visible and near-infrared regions, which is why these molecules are used for photodynamic therapy for cancer [17,18].

Several studies based on MPc have been conducted in chemical sensing. The results revealed that by varying the central metal ions of the MPc, the ionization potential, optical properties, sensitivity, and selectivity could change when in contact with different gas molecules. For example, Rossignol et al. [19] studied the detection of the NH_3_ and O_3_ gases in the range of 20 to 200 ppm using a cobalt phthalocyanine sensor. Moreover, there has been a lot of research conducted on the detection of NO_2_ and NH_3_ with MPcs (M = Pd, Co, Cu, Mg, Ti, Ni, and Zn) [20–24]. It is important to mention that a well-known phthalocyanine complex of titanium and vanadium is the metal-oxo complex, with a M^4+^ redox state (M = Ti and V) [25,26]. However, under some particular chemical conditions, low-valent metal species could be stable. For example, Eguchi et al. [27], in their experimental investigation, synthesized the VPc monolayer on the Ag (111) under ultra-high-vacuum condition. The variation of the axial ligands, the functional groups of the side chains, and µ-bridging atom is able to induce different electronic properties to the MPc complexes. Consequently, by designing and optimizing these synthesized thin films, it is possible to produce MPcs with different degrees of sensitivity, selectivity, and stability. In this regard, Blowey et al. [28], in their valuable study, demonstrated that vanadyl phthalocyanine (VOPc) can stabilize on the surface of Cu (111) from the up and down orientations of the V = O moiety. Moreover, the surface of the VPc complex (VOPc without axial oxygen) was actually available in the down orientation of the VOPc complex. Lu et al. [29] conducted a systematic study of the ability of the porous sheet of MPcs (Sc to Zn) toward hydrogen storage. They concluded that the ScPc sheet was able to store 4.6% hydrogen and might be a promising material for H_2_ storage. However, there are a few studies on the application of MPc complexes (M = Sc, Ti, and V).

The ability of transition MPcs in the interaction with different gases, on one side, and the existence of the CH_2_O molecule in ambient air as a pollutant, on the other side, provided the motivated herein to study the interaction of MPc (M = Sc, Ti, and V) complexes with FA gas. Arokiyanathan et al. [30] showed that the substitution of functional groups did not affect the properties of the MPc ring (Sc and Mg). Accordingly, to reduce the computational cost, the effect of the axial groups, functional groups, and substrates that were present in many synthesized MPc complexes were not considered herein. All in all, in the current work, the electronic properties and adsorption behaviors of the [MPc]^a^ (M = Sc, Ti, and V; a = –1, 0, 1) complexes toward the FA molecule were studied. The interaction of H2CO with the three considered forms of the [MPc]^a^ (–1, 0, and +1) complexes was investigated based on analyses of the preferable orientations, electronic properties, and corresponding binding energies within the density functional theory (DFT) framework.

## 2. Computational details

All of the calculations performed herein were conducted using the Kohn-Sham DFT with the Becke three-parameter hybrid, non-local exchange, and correlation functional, known as B3LYP [31,32], in conjunction with the split-valence basis set 6-31G* for the H, N, C, and O atoms, and the LANL2DZ basis set for the transition metals, as implemented in the Gaussian 09 program (Gaussian, Inc., Wallingford, CT, USA) [33]. Moreover, previous studies revealed that the use of the B3LYP functional performs reasonably well to predict ground-state properties and gas adsorption on the MPc complexes [34–38]. The validity of the global minimum was determined using the calculation of the vibrational frequencies, in which the absence of vibrational modes with imaginary frequencies demonstrated that the optimized molecular geometry was found at the lowest energy structure. The values of the atomic charges were estimated by employing natural bond orbital (NBO) analysis with the same geometry optimization level. In addition, to obtain accurate results for adsorption of the gases over the MPcs, and taking into account the Van der Waals forces, the results were obtained by considering the long-range correction scheme introduced by Grimme (i.e. the D3-DFT method) [39]. 

The zero-point energy (ZPE) corrected interaction energy ( *E* *int* ) for the complexes were calculated by employing the following formula:

(1)Eint=(EGas-PMc-EPMc-EGas)+(EGas-PMcZPE-EPMcZPE-EGasZPE)+BSSE

Here, *E*_*Gas* ‒ *PMc*_ is the total energy of [Gas/MPc] complex, and *E*_*PMc*_ and *E*_*Gas*_ are the calculated energies for the pure MPc complex and the gas molecule, respectively. Based on the obtained results, a negative value of *E*_*int*_ showed that the adsorption process had an exothermic character. Furthermore, the basis set superposition error was eliminated by taking into account the standard counterpoise correction method of Boys and Bernardi [40].

the highest occupied molecular orbital (HOMO) and lowest unoccupied molecular orbital (LUMO) energy gap (HOMO–LUMO) ( *E*_*g*_ ) was obtained using the following equation: 

(2)Eg=εLUMO-εHOMO

where ε_*LUMO*_ and ε_*HOMO*_ are the corresponding energies of the LUMO and HOMO, respectively. 

The change in the HOMO-LUMO energy gap (Δ *E*_*g*_) was calculated as in Eq. (3) for the [MPc]^a^ (M = Sc, Ti, and V; a = –1, 0, and +1) complexes toward the FA adsorption.

(3)ΔEg=|[(Eg2-EG1)]|x100

Here, *E*_*g1*_ and *E*_*g2*_ are the values of *E*_*g*_ in the initial (bare [MPc]^a^) and final state ([gas/MPcs]^a^ complexes), respectively.

## 3. Results 

### 3.1. Electronic structure of the [ScPc]^a^ (a = –1, 0, and +1) complexes

**Figure 1:**
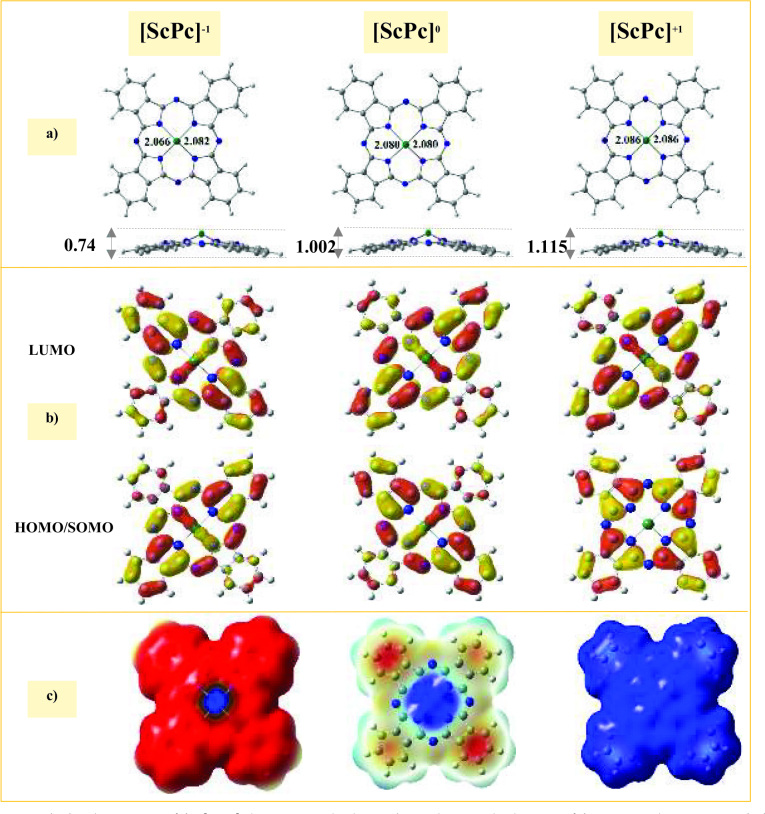
Calculated properties of the [ScPc]^a^ (a = −1, 0, and +1) complexes. a) Top and side views of the optimized structures with the bond distances in Å, b) SOMO/HOMO and LUMO spatial distributions with an isovalue of 0.02 e/Å^3^, and c) molecular electrostatic potential isosurface (0.0004 au). The C, H, N, and Sc atoms are shown in grey, white, blue, and green, respectively.

The optimized structures of the [ScPc]^a^ (a = –1, 0, and +1) complexes are shown in Figure 1a. The data analyses showed that the ScPc in all three of the oxidized, neutral, and reduced states had a nonplanar geometric structure with C2v symmetry. Moreover, the Sc atom was projected by about 1.115, 1.002, and 0.774 Å out of the molecular planes of the [ScPc]+1, [ScPc]0, and [ScPc]−1 complexes, respectively. It was found that increasing the negative charge on the ScPc complex reduced the out-of-plane projection of the Sc atom. The calculated Sc-N bond lengths were 2.086, 2.080, and 2.082 Å for the [ScPc]+1, [ScPc]0, and [ScPc]−1 complexes, respectively. These data also showed an increase of 0.006 and 0.002 Å in the Sc-N bond length in the oxidized and reduced states, which showed that these bonds were weaker than that of neutral state. Moreover, the calculated bond orders for the Sc-N bond were 0.73 and 0.45 for the [ScPc]^-1^ and [ScPc]^0^ states, respectively, which demonstrated that this bond order decreased by inducing the positive charge on the ScPc complexes. Furthermore, increasing the positive charge on the ScPcs prevented the bond between the Sc and N atoms. In this regard, [ScPc]^+1^ may be an unstable and more reactive complex. 

Metal coordination is an important feature of the MPcs toward gas adsorption. In these structures the positively-charged M atoms coordinated to the four isoindole nitrogens of the phthalocyanine ring through covalent and electrostatic interactions. According to the NBO population analysis (see Table 1), the Sc atom presented a positive charge, with values of 1.89, 1.81, and 1.75 *e* for the [ScPc]^+1^, [ScPc]^0^, and [ScPc]^-1^ complexes, respectively. The values of the charge on the Sc atom revealed that, when the ScPc complex was reduced, a part of the induced charge remained close to the Sc atom.

**Table 1:** Spin multiplicity (2S+1), SOMO1/HOMO energy (E_SOMO/HOMO_, in eV), LUMO energy (E_LUMO_, in eV), SOMO-LUMO energy gap (E_g_, in eV), and NBO charge on the M atom (qM) for the bare [MPcs]^a^ (M = Sc, Ti, and V; a = –1, 0, and +1) complexes.

[MPc]	2S+1	Eg	ESOMO/HOMO	ELUMO	qM
[ScPc]−1	1	1.26	–0.96*	0.30	1.75
[ScPc]^0^	2	1.18	–4.03	–2.85	1.81
[ScPc]^+1^	1	1.82	–7.86*	–6.04	1.89
[TiPc]−1	4	2.20	–1.13	1.07	1.15
[TiPc]^0^	3	2.37	–4.79	–2.42	1.37
[TiPc]^+1^	2	1.58	–7.97	–6.39	1.42
[VPc]−1	5	2.17	–1.23	0.94	0.82
[VPc]^0^	2	2.34	–5.13	–2.79	0.96
[VPc]^+1^	3	1.19	–8.32	–7.12	1.06

* Values with asterisks are related to the energy of HOMO. ^1^ SOMO: singly-occupied molecular orbital.

Frontier molecular orbitals (FMOs) are important descriptors used to better understand the stability and reactivity of the complexes. In the electrophilic attack, the ionization potential was related to the HOMO. In the nucleophilic reactions, electronic affinity, and susceptibility were directly related to the LUMO. Moreover, the HOMO–LUMO energy gap ( *E* g) can be utilized to estimate the chemical stability and reactivity of the complexes. The spatial distribution of the FMOs of the ScPcs are presented in Figure 1b, where it can be seen that the electron density of the HOMO and LUMO orbitals was uniformly distributed over the surfaces of the [ScPc]^-1^ and [ScPc]^0^ complexes. However, the HOMO of the [ScPc]^+1^ complex was mainly localized on the phthalocyanine ring, without any contribution by the Sc atom. The *E* g values of the [ScPc]^+1^, [ScPc]^0^, and [ScPc]^-1^ complexes were 1.82, 1.18, and 1.26 eV, respectively. Therefore, as a result of the narrow HOMO-LUMO gap, the [ScPc]^0^ complex was more reactive than its counterparts toward FA adsorption.

A map of electrostatic potential (MEP) is a physical descriptor to predict the active site of complexes upon nucleophilic and electrophilic attacks, where the positive and negative electrostatic potential regions are represented by the suitable part of a complex in electrophilic and nucleophilic attacks, respectively. The MEP of the ScPcs is shown in Figure 1c, which demonstrates the distribution of negative and positive electrostatic potential regions on the ScPcs. Moreover, in all of the complexes, the positive region (in blue) is mainly localized on the Sc atom. Thus, the Sc atom had more potential to participate in the nucleophilic attack.

### 3.2. Electronic structure of the [TiPc]a (a = –1, 0, and +1) complexes

**Figure 2:**
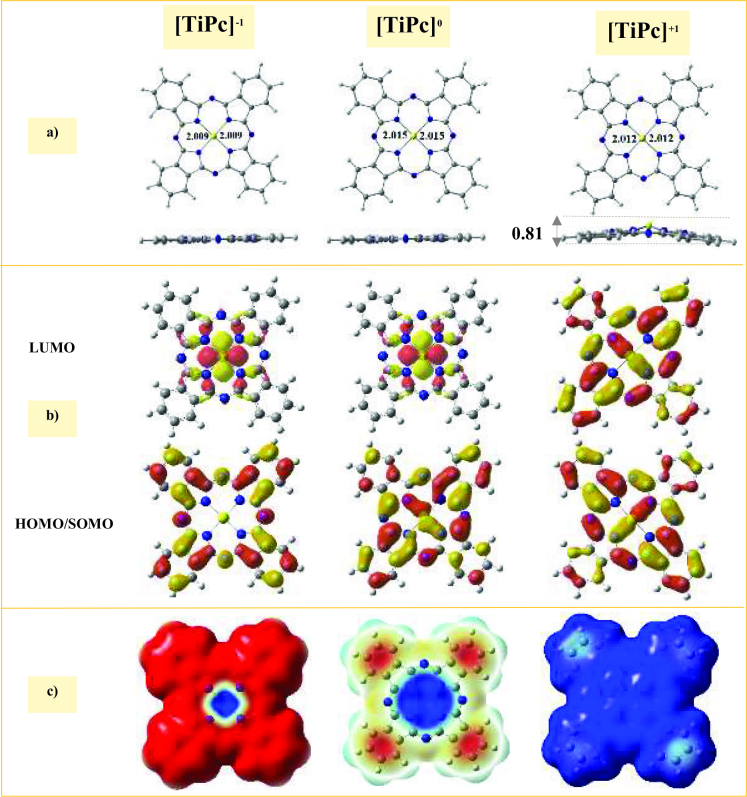
Calculated properties of the [TiPc]^a^ (a = −1, 0, and +1) complexes. a) Top and side views of the optimized structures with the bond distances in Å, b) SOMO/HOMO and LUMO spatial distributions with an isovalue of 0.02 e/Å^3^, and c) molecular electrostatic potential isosurface (0.0004 au). The C, H, N, and Ti atoms are shown in grey, white, blue, and yellow, respectively.

The optimized structures of the [TiPc]^a^ (a = –1, 0, and +1) complexes are shown in Figure 2a. Although both the [TiPc]^-1^ and [TiPc]^0^ complexes were completely planar, the [TiPc]^+1^ complex had a nonplanar structure, in which the Ti atom was about 0.814 Å out of the molecular plane. The calculated Ti-N bond distances were 2.012, 2.015, and 2.009 Å for the [TiPc]^+1^, [TiPc]^0^, and [TiPc]^-1^ complexes, respectively. These results showed that the Ti-N bond lengths in the reduced and oxidized states were 0.006 and 0.003 Å less than in the neutral state, which showed that this bond was stronger than that of their neutral counterparts. 

The NBO analysis revealed that there was no actual bond between the Ti and N atoms in any of the three considered TiPc complexes, which explained why the isolated TiPc complex was not observed experimentally. Accordingly, to stabilize the TiPc complexes, it is important to use a substrate. However, the oxo-TiPc adsorbed from the O-head on a substrate or the directly synthesized TiPc on a surface may be used as a bare TiPc complex in practical applications. The base on the NBO analysis (see Table 1) and the titanium atoms present positive charges, at values of 1.42, 1.37, and 1.15 *e* in the [TiPc]^+1^, [TiPc]^0^, and [TiPc]^-1^ complexes, respectively, were due to the fact that a part of the induced charge remained close to the Ti atom after reducing the TiPc complex. 

According to the spatial distribution of the FMOs of TiPc complexes (see Figure 2b), the LUMOs of [TiPc]^-1^ and [TiPc]^0^ complexes were more localized on the Ti atom, which indicated that when these complexes were attacked by a nucleophilic compound, the electrons went to the *d* orbital of the Ti atom. This was further confirmed by the MEP plots, where the positive charges were distributed across the Ti atom and created an electric field on the surface of the [TiPc]^-1^ and [TiPc]^0^ complexes (see Figure 2c). This may lead to the polarization of the incoming gas molecules during nucleophilic attacks.

The *E* g values of the [TiPc]^+1^, [TiPc]^0^, and [TiPc]^-1^ complexes were 1.58, 2.37, and 2.20 eV, respectively. Accordingly, the reactivity of the TiPc complexes toward gas adsorption decreased in the order of [TiPc]^+1^>[TiPc]^-1^>[TiPc]^0^. 

### 3.3. Electronic structure of the [VPc]^a^ (a = –1, 0, and +1) complexes

**Figure 3:**
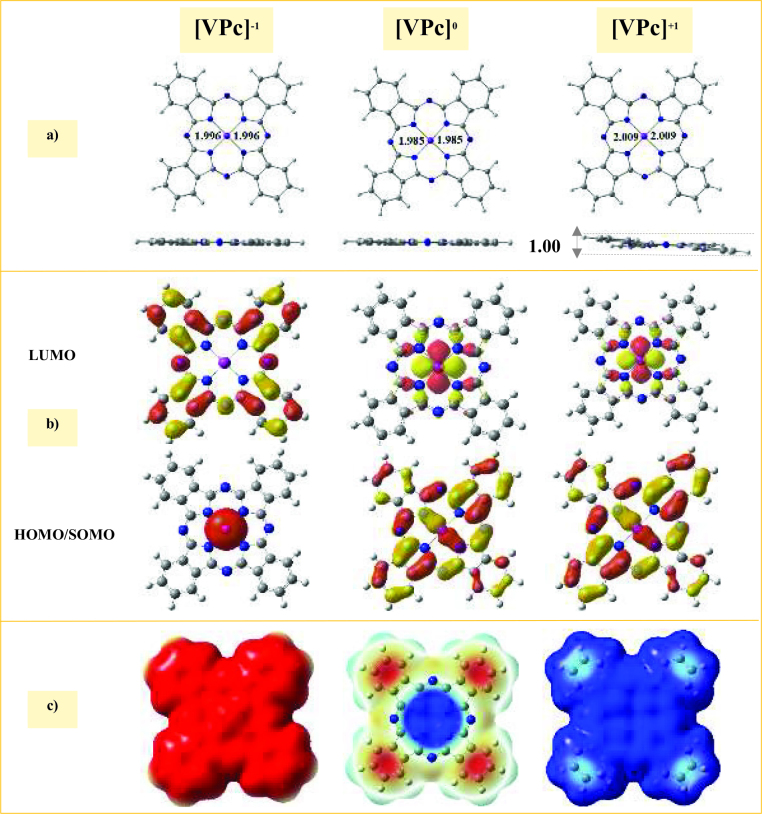
Calculated properties of the [VPc]^a^ (a = −1, 0, and +1) complexes. a) Top and side views of the optimized structures with the bond distances in Å, b) SOMO/HOMO and LUMO spatial distributions with an isovalue of 0.02 e/Å^3^, and c) molecular electrostatic potential isosurface (0.0004 au). The C, H, N, and V atoms are shown in grey, white, blue, and violet, respectively.

The optimized [VPc]^-1^ and [VPc]^0^ complexes were completely planar (see Figure3a), while the structure of the [VPc]^+1^ complex had a curvature, and one of its sides was about 1.001 Å higher than the other side. The calculated V-N bond lengths were 2.009, 1.999, and 1.996 Å in the [VPc]^+1^, [VPc]^0^, and [VPc]^-1^ complexes, respectively. These results showed that the V-N bond lengths in the oxidized and neutral states were 0.010 and 0.013 Å higher than in the reduced state, which showed that this bond was weaker than that of their reduced counterparts. 

The NBO population analysis (see Table 1) showed that the vanadium atom presented a positive charge, with values of 1.06, 0.96, and 0.82 *e* in the [VPc]^+1^, [VPc]^0^, and [VPc]^-1^ complexes, respectively. Clearly, the charge in the V atom was more altered with the induction of both the positive and negative charges, in which a part of the induced charge remained close to the V atom.

Based on the spatial distribution of the FMOs (see Figure 3b), it was observed that the LUMOs of the [VPc]^0^ and [VPc]^+1^ complexes protruded more from the molecular plane, which indicated that the V atom of these complexes can be used as a target site under nucleophilic attacks. In the case of the [VPc]^-1^ complex, the HOMO was mainly localized on the V atom, which demonstrated that when an electron was extracted from this complex, the extracted electron was from the V atom. The negative electrostatic potential was mainly localized on the whole surface of the [VPc]^-1^ complex, which suggested that the [VPc]^-1^ complex may be inert under nucleophilic attacks (see Figure 3c). The *E* g values of the [VPc]^+1^, [VPc]^0^, and [VPc]^-1^ complexes were 1.19, 2.34, and 2.17 eV, respectively. It was predicted that the [VPc]^+1^ complex may be more reactive than its counterparts towards gas adsorption.

### 3.4. Formaldehyde adsorption on the metal phthalocyanine complexes ([MPc]^a^, M = Sc, Ti, and V; a = –1, 0, and +1)

Based on the results, the calculated C-H bond length and H-C-H bond angle of the free CH_2_O was 1.11 Å and 115.2°, which was in good agreement with the reported experimental data of 1.01 Å and 118°, respectively [41]. These results suggested that the theoretical method used in this study was accurate to describe the studied system. 

In order to obtain the stable structure of the CH_2_O adsorbed on the surface of the MPcs, several possible initial adsorption geometries were considered, including the hydrogen site, oxygen site, and CH_2_O surface perpendicular or parallel to the surface of the MPcs. However, the adsorption of the CH_2_O from the O-side led to a more stable structure that was in good accordance with other theoretical and experimental studies [42–44].

**Figure 4:**
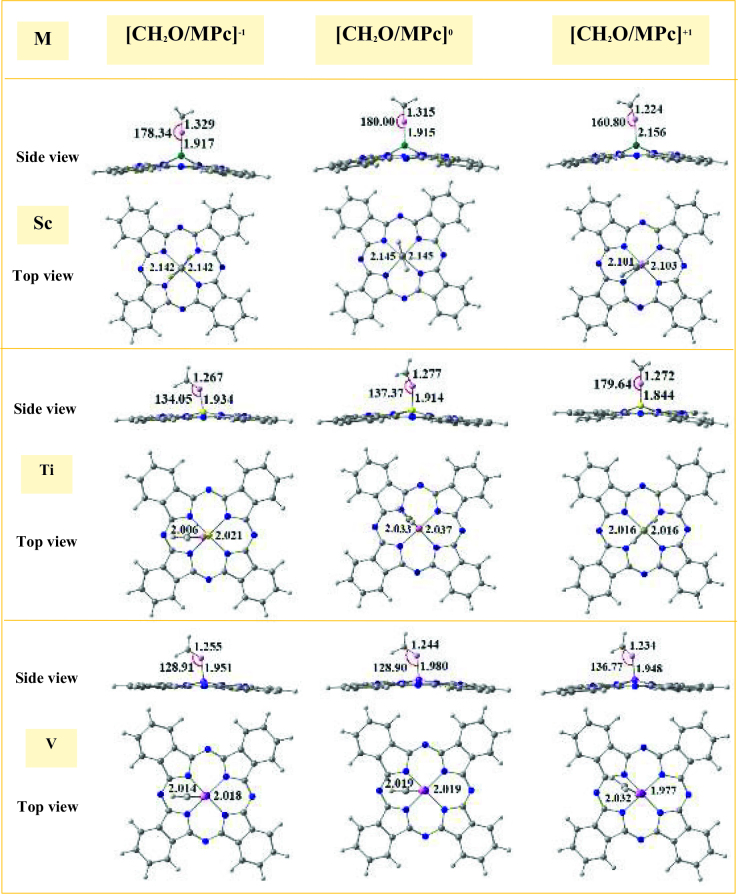
Top and side views of the optimized structures of the [CH_2_O/MPc]^a^ (M = Sc, Ti, and V, a = −1, 0, and +1) complexes with the bond distances in Å and bond angle in degrees. The C, H, N, O, Sc, Ti, and V atoms are shown in grey, white, blue, pink, green, yellow, and violet, respectively.

Figure 4 shows the calculation results, where it can be seen that the Sc-O bond lengths in the reduced and oxidized states were 0.002 and 0.241 Å less than that of the neutral state. It was obvious that the [ScPc]^+1^ complex had less tendency to adsorb the FA gas in comparison with the other studied Sc complexes. Moreover, the projection of the Sc atom from the plane of the [CH_2_O/ScPc]^a^ complex was ~0.17 Å smaller than that of the bare clusters. This clearly demonstrated that the adsorption of CH_2_O molecule over the three considered forms of the [ScPc]^a^ complexes helped to stabilize these complexes.

In the optimized structure of the [CH_2_O/TiPc]^a^ complexes (Figure 4), the Ti-O bond distances were found as 1.934, 1.914, and 1.844 Å for the [TiPc]^-1^, [TiPc]^0^, and [TiPc]^+1^ complexes, respectively. Therefore, it can be inferred that CH_2_O was strongly bonded to the Ti atom in the oxidized state when compared to its counterparts. Furthermore, the main change that happened during the CH_2_O adsorption over the [TiPc]^-1^ and [TiPc]^0^ complexes was the projection of the Ti atom out of the molecular plane of these complexes (~0.15 Å).

Figure 4 also displays the optimized structure of the [CH_2_O/VPc]^a^ complexes. Based on these results, the V-O bond distances were 0.003 and 0.032 Å longer in the reduced and neutral states when compared to the oxidized state of the VPc, respectively. Additionally, the V atom remained about 0.12 Å out of the plane of the [CH_2_O/VPc]a complexes. 

It is important to mention that other optimized structure parameters, such as the M-N and N-C bond lengths, did not change dramatically after CH_2_O adsorption over the studied complexes, due to the interaction of CH_2_O with only the metal ion of the MPcs. Moreover, the Pc molecules only had a role as host for the M atom and created an avenue for the charge transfer during adsorption.

The partial charges of the M atom on the CH_2_O/MPcs complexes derived from the NBO analysis are tabulated in Table 2, where it can be seen that the adsorption of CH_2_O on the MPcs led to a strong charge transfer between the M atom of MPcs, and oxygen atom of the CH_2_O molecule. Generally, the positive charges on the M (Sc, Ti, and V) atom of the [CH_2_O/MPc] complexes increased by increasing the positive charge on the whole complex. As can be seen in Figure 4, this charge transfer to the FA gas was closely related to variations of the M-O-CH2 angle, which demonstrated two conformations, i.e. bent or linear. The concentration of the negative charge in the FA characterized linear geometry (∠M-O-CH_2_ = 179.50°) and was due to the strong interaction between the M atom and CH_2_O molecule.

**Table 2:** SOMO^1^/HOMO energy (E_SOMO/HOMO_, in eV), LUMO energy (E_LUMO_, in eV), SOMO/HOMO-LUMO energy gap (Eg, in eV), NBO charge on the M atom in [(CH_2_O)MPc] complexes (qM), NBO charge transfer from [CH_2_O/MPc]^a^ complexes to the CH_2_O molecule (Q), and the absolute value of the change in the Eg (|∆Eg|, %) for the [CH_2_O/MPcs]^a^ (M = Sc, Ti, and V; a = –1, 0, and +1) complexes.

[(CH_2_O)MPc]	2S + 1	E_g_	E_SOMO/HOMO_	E_LUMO_	E_int_	q_M_	Q	ΔE_g_
[CH_2_O/ScPc]^-1^	3	1.06	–0.86	0.20	–1.51	+1.56	–0.24	16
[CH_2_O/ScPc]^0^	2	1.15	–4.00	–2.85	–1.55	+1.61	–0.45	2
[CH_2_O/ScPc]^+1^	1	0.95	–7.59*	–6.63	–1.25	+1.69	–0.19	48
[CH_2_O/TiPc]^-1^	2	1.74	–1.10	0.63	–0.87	+1.20	–0.17	21
[CH_2_O/TiPc]^0^	1	1.52	–4.32*	–2.80	–1.09	+1.27	–0.15	36
[CH_2_O/TiPc]^+1^	4	1.32	–7.18	–5.86	–1.01	+1.31	–0.23	16
[CH_2_O/VPc]^-1^	3	2.13	–1.68	0.45	–0.64	+0.80	0.02	2
[CH_2_O/VPc]^0^	4	2.23	–4.89	–2.66	–2.09	+0.82	0.07	5
[CH_2_O/VPc]^+1^	3	1.26	–7.75	–6.48	–1.06	+0.93	–0.10	6

* Values with asterisks are related to the energy of HOMO.

In addition, vibration frequency analysis showed that the vibration mode of C-O bond in the free CH_2_O molecule was about 1849 cm−1, while it was significantly red-shifted to ~1261 cm−1 after CH_2_O adsorption over the Sc and Ti complexes. It is worth mentioning that the charge was transferred from these molecules to the formally empty *π* ∗ orbital of the C-O bond and consequently, weakened this bond. At the same time, *π* -back bonding occurred from these complexes to the C-O bond. The calculated C-O bond length was about 1.11 and ~1.25 Å in the free CH_2_O and [CH_2_O/MPc]^a^ (M = Sc, Ti, and V) complexes, which u1e70 the weakening of the C-O bond after adsorption.

The interaction strength of the [MPc]^a^ complexes and CH_2_O molecule can be further evaluated by calculating the interacting energy that this interaction led to in the formation of the [CH_2_O/MPc]a complexes. The general reaction of the complex formation can be written as:

[MPc]^a^+ CH_2_O → [(CH_2_O)MPc]^a^ a = –1, 0, and +1.

The energy values obtained from the calculations of the CH_2_O complexation with the [ScPc]^-1^, [ScPc]^0^, and [ScPc]^+1^ states were –1.51, –1.55, and –1.25 eV, respectively (see Table 2). These values showed that the CH_2_O molecule was strongly bonded to [ScPc]^0^ complex when compared to the [ScPc]^-1^ and [ScPc]^+1^ states. As a result of the large negative *E*_*int*_ for the [ScPc]^a^ complexes, chemical adsorption may occur during CH_2_O interaction with the [ScPc]^a^ complexes. In the case of CH_2_O complexation with the [TiPc]^-1^, [TiPc]^0^, and [TiPc]^+1^ states, the interaction energy values were found as –0.87, –1.09, and –1.01 eV, respectively. These results showed that the interaction between CH_2_O and the reduced form of the TiPc complexes was weak. The *E*_*int*_ values were –0.64, –2.09, and –1.06 eV for the [CH_2_O/VPc]−1, [CH_2_O/VPc]0, and [CH_2_O/VPc]+1 complexes, respectively. As a result, the adsorption of CH_2_O on the [CH_2_O/VPc]−1 complex was weak. These results were further supported by the negligible charge transfer between CH_2_O and the [CH_2_O/VPc]^-1^complex (see Table 2). 

**Figure 5:**
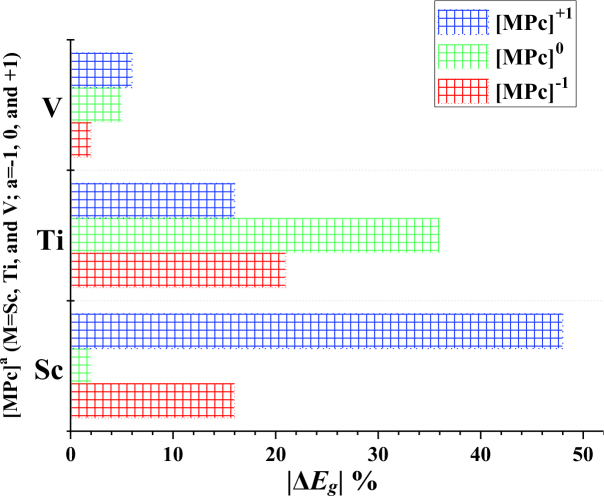
Absolute value of the change in the E_g_ (ΔE_g_) for the [MPcs]^a^(M = Sc, Ti, and V, a = −1, 0, and +1) complexes.

**Figure 6:**
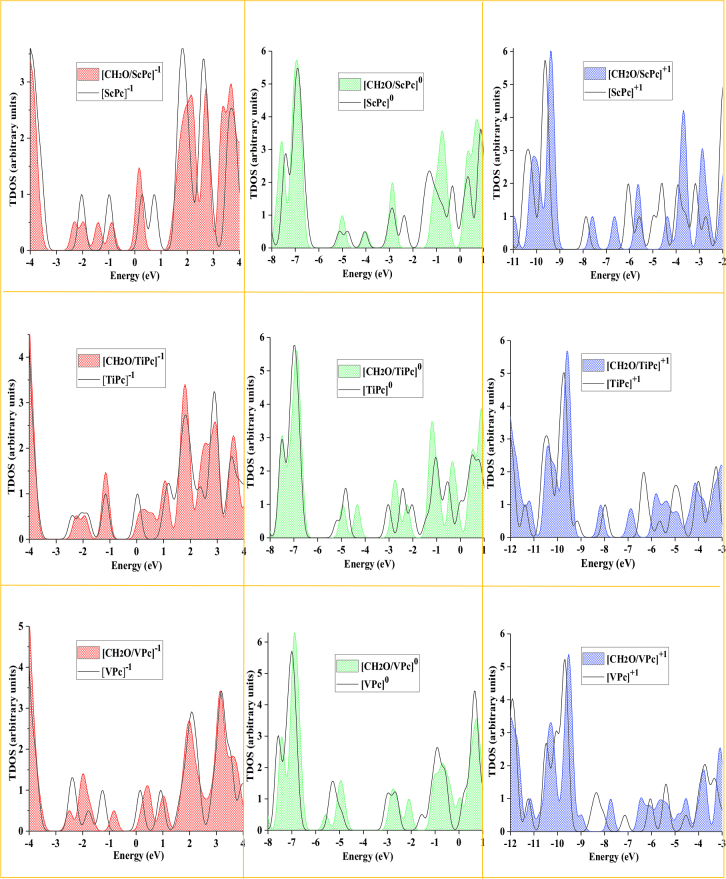
TDOS plots for the [MPc]a complexes (black curves) and [CH_2_O/MPc]^a^ (M = Sc, Ti, and V, a = −1, 0, and +1) complexes (color-filled curves).

In experimental studies, the variation in elctrical conductivity after gas adsorption was measured as a sign of the sensitivity of an electronic sensor toward a specific molecule [45,46]. In theoretical studies, the change of *E*_*g*_ was considered as a characteristic of the sensor to simulate electrical conductivity [47,48]. In this regard, the ScPc and TiPc complexes that showed a decrease in *E*_*g*_ after CH_2_O adsorption could demonstrate high electrical conductivity. In other words, by tracing the u1e97 variation of the ScPc and TiPc complexes before and after CH_2_O adsorption, the presence of this molecule can be detected in the air (see Figure 5). Moreover, among the studied forms of the MPc complexes, it was determined that the [ScPc]^+1^ and [TiPc]^0^ complexes can be a promising CH_2_O sensing material due to the significant decrease in *E*_*g*_ after CH_2_O adsorption. This result was confirmed by the total density of the state plots (TDOS) of CH_2_O adsorption over the MPc complex (see Figure 6), where the appearance of a new peak between the HOMO and LUMO after adsorption led to a decrease in the *E*_*g*_ value of the [ScPc]^+1^ and [TiPc]^0^ complexes. In addition to these results, Figure 4a indicated that, in the case of [ScPc]^0^, the newly formed Sc-O bond in comparison with other M-O bonds in the neutral form of MPcs was short (1.915 Å), but the *E*_*g*_ value (see Table 2) after adsorption changed somewhat (~0.03 eV), due to significant structural distortion. Moreover, the adsorption of CH_2_O over the vanadium complexes did not alter the *E*_*g*_ value, due to the distortion of the structure of the VPc complexes after CH_2_O adsorption. It is important to mention that, the calculations herein were 298.15 K and 1 atm. However, the calculation results at other temperatures, pressures, and FA concentrations might be different, which should be taken into consideration by experimental and theoretical researchers in future studies.

## 4. Conclusions

First-principle calculations were performed to study the adsorption and interaction of the CH_2_O molecule toward a variety of [MPc]^a^ (M = Sc, Ti, and V; a = –1, 0, and +1) complexes. It was found that the adsorption of CH_2_O over the MPCs was able to significantly alter the electronic properties of the Sc and Ti complexes. The results of the present study revealed that the most pronounced effect on the electronic structure of the MPcs was observed with [ScPc]^+1^, where the *E*_*g*_ value of [ScPc]^+1^ showed a variation of about 48% after CH_2_O adsorption. It is expected that the present study will stimulate experimental efforts to confirm the results of this work as well as devise chemical modification strategies to design effective MPcs-based gas sensors by employing MPc (M = Sc, Ti, and V) complexes.

## References

[ref1] (1996). Modification of silver catalysts for oxidation of methanol to formaldehyde. Catalysis Today.

[ref2] (1986). Conversion of methanol, formaldehyde and formic acid on the polar faces of zinc oxide. Surface Science.

[ref3] (2007). Functionalized metallic carbon nanotube devices for pH sensing. ChemPhysChem.

[ref4] (2006). Single nanoporous gold nanowire sensors. The Journal of Physical Chemistry B.

[ref5] (2009). Biomolecule‐functionalized nanowires: from nanosensors to nanocarriers. ChemPhysChem.

[ref6] (2005). Silicon nanowire sensors for bioanalytical applications: glucose and hydrogen peroxide detection. Advanced Functional Materials.

[ref7] (2005). Fabrication and application of long strands of silicon nanowires as sensors for bovine serum albumin detection. Applied Physics Letters.

[ref8] (2004). Polymeric nanowire chemical sensor. Nano Letters.

[ref9] (2000). Nanoparticle arrays on surfaces for electronic, optical, and sensor applications. ChemPhysChem.

[ref10] (2010). Monolayer-capped cubic platinum nanoparticles for sensing nonpolar analytes in highly humid atmospheres. The Journal of Physical Chemistry C.

[ref11] (2007). Porous metal oxides as gas sensors. Chemistry–A European Journal.

[ref12] (2018). Fluorinated metal phthalocyanines: interplay between fluorination degree, films orientation, and ammonia sensing properties. Sensors.

[ref13] (2016). Electrocatalytic oxidation of Epinephrine and Norepinephrine at metal oxide doped phthalocyanine/MWCNT composite sensor. Scientific Reports.

[ref14] (2017). Sensing of volatile organic compounds by copper phthalocyanine thin films. Materials Research Express.

[ref15] (2001). Order on disorder: Copper phthalocyanine thin films on technical substrates. Journal of Applied Physics.

[ref16] (2014). Fluorination of metal phthalocyanines: Single-crystal growth, efficient n-channel organic field-effect transistors, and structure-property relationships. Scientific Reports.

[ref17] (2014). Oncologic photodynamic diagnosis and therapy: confocal Raman/fluorescence imaging of metal phthalocyanines in human breast cancer tissue in vitro. Analyst.

[ref18] (2001). Current status of phthalocyanines in the photodynamic therapy of cancer. Journal of Porphyrins and Phthalocyanines.

[ref19] (2011). Differential study of substituted and unsubstituted cobalt phthalocyanines for gas sensor applications. Sensors and Actuators B: Chemical.

[ref20] (2009). Comparative gas sensing in cobalt, nickel, copper, zinc, and metal-free phthalocyanine chemiresistor. Journal of the American Chemical Society.

[ref21] (2017). Thin films of unsubstituted and fluorinated palladium phthalocyanines: Structure and sensor response toward ammonia and hydrogen. The Journal of Physical Chemistry C.

[ref22] (2017). Tetrasubstituted copper phthalocyanines: correlation between liquid crystalline properties, films alignment and sensing properties. Sensors and Actuators B: Chemical.

[ref23] (2002). Conducting polymers functionalized with phthalocyanine as nitrogen dioxide sensors. Sensors.

[ref24] (2017). High-sensitive room-temperature NO_2_ sensor based on a soluble n-type phthalocyanine semiconductor. Inorganic Chemistry Communications.

[ref25] (2001). Influence of humidity conditions on the capacitive and resistive response of an Al/VOPc/Pt co-planar humidity sensor. Measurement Science and Technology.

[ref26] (2007). The preparation of high photosensitive TiOPc. Dyes and Pigments.

[ref27] (2015). Direct synthesis of vanadium phthalocyanine and its electronic and magnetic states in monolayers and multilayers on Ag (111). The Journal of Physical Chemistry C.

[ref28] (2018). The Structure of VOPc on Cu (111): Does V═ O Point Up, or Down, or Both?. The Journal of Physical Chemistry C.

[ref29] (2011). Sc-phthalocyanine sheet: Promising material for hydrogen storage. Applied Physics Letters.

[ref30] (2018). Impact of functional groups substitution on the molecular properties of magnesium and scandium phthalocyanines. Inorganica Chimica Acta.

[ref31] Density-functional exchange-energy approximation with correct asymptotic behavior. Physical Review.

[ref32] (1988). Development of the Colle-Salvetti correlation-energy formula into a functional of the electron density. Physical review B.

[ref33] (01). Gaussian.

[ref34] (2015). Ground and excited states of zinc phthalocyanine, zinc tetrabenzoporphyrin, and azaporphyrin analogs using DFT and TDDFT with Franck-Condon analysis. The Journal of chemical physics.

[ref35] (2014). Fluorination of metal phthalocyanines: Single-crystal growth, efficient n-channel organic field-effect transistors, and structure-property relationships. Scientific Reports.

[ref36] (2019). New zinc phthalocyanine derivatives for nitrogen dioxide sensors: A theoretical optoelectronic investigation. Journal of Molecular Graphics and Modelling.

[ref37] (2012). Metallophthalocyanine and Metallophthalocyanine–fullerene complexes as potential dye sensitizers for solar cells DFT and TD-DFT calculations. Organic Electronics.

[ref38] (2014). Surface acoustic wave sensor based on nickel (II) phthalocyanine thin films for organophosphorous pesticides selective detection. Sensors IEEE.

[ref39] (2014). DFT-D3 study of some molecular crystals. The Journal of Physical Chemistry C.

[ref40] (1970). The calculation of small molecular interactions by the differences of separate total energies. Some procedures with reduced errors. Molecular Physics.

[ref41] (1997). Chemistry: the Molecular Science. Jones & Bartlett Learning.

[ref42] (2017). Formaldehyde adsorption on the anatase TiO2 (101) surface: experimental and theoretical investigation. The Journal of Physical Chemistry C.

[ref43] (2017). Adsorption/desorption process of formaldehyde onto iron doped graphene: a theoretical exploration from density functional theory calculations. Physical Chemistry Chemical Physics.

[ref44] (2019). A. Modulation of the electronic properties of pristine and AlP-codoped stanene monolayers by the adsorption of CH_2_O and CH4 molecules: a DFT stud. Materials Research Express.

[ref45] (2015). Graphene: highly sensitive and selective gas sensor. The Journal of Physical Chemistry C.

[ref46] (2018). Two-dimensional transition metal carbides and nitrides (MXenes) for biomedical applications. Chemical Society Reviews.

[ref47] (2019). Boron-decorated graphitic carbon nitride (g-C3N4): an efficient sensor for H2S, SO2, and NH_3_ capture. The Journal of Physical Chemistry C.

[ref48] (2017). Toward the realization of 2D borophene based gas sensor. The Journal of Physical Chemistry C.

